# 

**Published:** 1847-10

**Authors:** 


*Quack Advertisements.*—The Boston Chronotype, a daily newspaper, published at Boston, Mass., says, " our rule about the admission of advertisements has been from the first, not to admit any business which we consider in itself immoral." Happening to glance at this editorial remark. we had the curiosity to look through the columns of the paper, and in so doing we counted about a dozen advertisements of secret remedies, professing to cure without fail certain, or all diseases, having no reference to age, sex, and the numerous circumstances peculiar to individual cases. We are therefore to infer that in the opinion of the editor of that paper, it is a moral business to practice upon community impositions connected with the patent medicine system. He would decline notices for the sale of spirituous liquors, and, we presume, his conscience would not permit him to advertise for gambling houses, and lottery brokers; but he has no scruples in aiding to dupe the credulous, not out of their money only, but of health, and, perhaps life. Singular consistency! We do not wonder that the Cronotype contains quack advertisements, for there are but few perhaps, as yet, that are sufficiently disinterested to refuse them, and the pecuniary profit therewith connected. But the protestation we have quoted, taken in connection with the practice, is what we admire! The good in this world seems indissolubly connected with the bad. The press is a mighty engine for the advancement of civilization, but it is, also, a powerful instrument for evil, and has its multitude of sins to answer for.

---

[Several editorial notices in type are excluded.]

